# Editorial: Salt Tolerant Rhizobacteria: For Better Productivity and Remediation of Saline Soils

**DOI:** 10.3389/fmicb.2021.660075

**Published:** 2021-04-22

**Authors:** Naveen Kumar Arora, Dilfuza Egamberdieva, Samina Mehnaz, Wen-Jun Li, Isha Mishra

**Affiliations:** ^1^Department of Environmental Science, Babasaheb Bhimrao Ambedkar University, Lucknow, India; ^2^Leibniz Centre for Agricultural Landscape Research (ZALF), Müncheberg, Germany; ^3^Department of Biotechnology, Al-Farabi Kazakh National University, Almaty, Kazakhstan; ^4^School of Life Sciences, Forman Christian College (A Chartered University), Lahore, Pakistan; ^5^State Key Laboratory of Biocontrol, Guangdong Provincial Key Laboratory of Plant Resources and Southern Marine Science and Engineering Guangdong Laboratory (Zhuhai), School of Life Sciences, Sun Yat-Sen University, Guangzhou, China; ^6^Department of Microbiology, Babasaheb Bhimrao Ambedkar University, Lucknow, India

**Keywords:** saline soils, salt tolerant rhizobacteria, plant growth promoting rhizobacteria, bioremediation, agro-ecosystems

Soil salinity has been recognized as a major issue particularly in arid and semi-arid areas of the world and is one of the main constraints that undermine plant growth and agricultural productivity. The pace at which saline soils are increasing around the globe has posed a serious threat to food security, the environment, and biodiversity. The Research Topic entitled “Salt Tolerant Rhizobacteria: For Better Productivity and Remediation of Saline Soils” is focussed on reviews and research articles on major challenges caused by soil salinization in agroecosystems and its remediation using salt-tolerant rhizobacteria as sustainable solutions to increase the productivity of these degraded lands.

The electrical conductivity (EC) of saline soils is either equal to or exceeds 4dS/m. Saline soils have a higher concentration of the salts Na^+^, Cl^−^, Ca^2+^, HCO^3−^, Mg^2+^, NO^3−^, and ${\text{SO}}_{4}^{2-}$, which prove detrimental to the microbial communities of the soil. The problems associated with salt accumulation are visible in several vital functions of soil such as poor water holding capacity and structural stability, reduced infiltration rate, disturbed pH, decreased levels of nutritional content, and lower organic matter. A study by Wang et al. explored the soil factors determining the structure and composition of bacterial communities in saline soils of Songnen Plain, China to dig out the potential of microbial resources. Systemic analysis using high throughput sequencing (Illumina MiSeq sequencing) revealed that the EC of the soil is one of the direct environmental factors that control the distribution of bacterial communities. The above concept was also explained in another research article by Yang et al., in which a metagenomic approach and *NifH* Illumina sequencing has been carried out for the characterization of rhizosphere and nodule microbiomes of wild salt-tolerant soybean growing in saline-alkaline soils of China. The study provides a systematic and functional understanding of the plant root microbiome under saline-alkaline conditions. Yang and Sun, have presented a similar correlation by showing how changes in soil properties regulate soil fungal communities and affect their distribution patterns and ecological functions.

There are myriad impacts on crops grown in salt stress conditions which have been interestingly detailed in a review by Kumar et al.. Such crops show signs of nutrient deficiency, ionic toxicity, oxidative stress, reduced photosynthetic activity, and decreased germination rate resulting in lower agricultural productivity (Kumar et al.). To obviate this problem, there is a need to adopt methods that are organic in origin, cost effective, and above all, ensure environmental sustainability. In this context, plant growth promoting rhizobacteria (PGPR) especially salt-tolerant or halotolerant PGPR have been identified as potential tools to alleviate salinity stress by eliciting tolerance in crops against high salt concentrations.

The mini review by Bhat et al., explains, salinity stress and plant productivity can be managed by halotolerant PGPR. The manuscript describes that halotolerant PGPR, due to their catabolic versatility and efficient root colonizing abilities, help in rejuvenating plant health through an array of mechanisms. These include nutrient acquisition, metal chelation, maintenance of water balance and ionic homeostasis, the production of phytohormones, exopolysaccharides (EPS), volatile organic compounds (VOCs), and antioxidative enzymes, triggering stress responsive genes under high salt concentrations. The article by Kaushal has reviewed similar aspects of PGPR by stating that microbially inoculated plants show well-established salt tolerance and endurance mechanisms (STEM), which include the aggregation of osmolytes, activating antioxidant machinery, recovery of nutritional status, and ionic homeostasis protecting the symbiotic partner under salt/ osmotic stress conditions. This theory was supported in a study by Nawaz et al., where they determined the potential of PGPR inoculation on two wheat genotypes (Aas-11; salt tolerant and Galaxy-13; salt sensitive). The outcome of the study revealed that PGPR inoculation significantly enhanced the physio-chemical attributes in both salt-tolerant and salt-sensitive wheat genotypes. In the same context, Singh et al., in their study showed that seeds bioprimed with selected strains, both individually or in combination, conferred better germination and vigor in maize plants. The authors showed that there is microbe based stress amelioration in maize plants making them ecologically fitter to survive and grow in saline-sodic soils.

A study by Taj and Challabathula also suggests that PGPR can increase the level of photosynthetic electron transport rate, and enhance carboxylation efficiency in their host plants during salinity stress. Recently, molecular tools have helped us to better understand the mode of actions of salt tolerant PGPR. Work by Nawaz et al., demonstrates the impact of acyl homoserine lactone (AHL) (bacterial signal molecules) producing PGPR *Aeromonas* sp., on two wheat genotypes. The study revealed that the exogenous application of AHL improved root parameters in both salt-sensitive and salt-tolerant genotypes. Principle component analysis (PCA) also showed the effectiveness of inoculation response of AHL producing *Aeromonas* in comparison to non-producing strains. The study opens a gateway for future exploration of AHL producing PGPR to exploit their role in the alleviation of salt stress and plant growth improvement. A comprehensive understanding of the complexities of signal transduction pathways can give new insights into biochemical and molecular mechanisms in response to salinity stress.

The beneficial characteristics of halotolerant PGPR make them excellent green solutions to enhancing the productivity of crops in saline agro-ecosystems in a sustainable manner. In the manuscript presented by Meena et al., the impact of inoculation of a halotolerant methylotrophic actinobacterium (*Nocardioides* sp. NIMMe6; LC140963) and the seed coating of its phytohormone-rich bacterial culture filtrate extract (BCFE) on wheat seedlings was investigated. The results suggested that bacterial inoculation mitigated saline stress in plants whereas seed priming with BCFE improved physiological status, enhanced oxidative enzymes, and resulted in gene modulation. The complete profiling of metabolites and the genes involved (of both the symbiotic partners) at inter and intra-cellular level will pave way for the development of reliable products for salt affected agro-ecosystems.

Salt specific metabolites and gene triggers will also be explored in the near future, as researchers explore tailor made halotolerant PGPR based bioinoculants. Vaishnav et al. examined the role of salt-tolerant PGPR *Sphingobacterium* BHU-AV3 on tomato plants under 200 mM NaCl concentration. The inoculated plants showed decreased levels of oxidative stress, lipid peroxidation, ROS, and cell death and enhanced levels of antioxidant enzymes and energy metabolism thus suggesting an overall plant protection strategy under salt stress. The potential of halotolerant PGPR under salt stress has also been reported on legumes. The research article by Alexander et al. shows that the inoculation of *Arachis hypogaea* by halotolerant PGPR *Stenotrophomonas maltophilia* BJ01 improved its photosynthetic pigments, auxin levels, and total amino acid content in salt stress conditions (100 mM NaCl) as compared to untreated plants.

The role of plant-microbiome under diverse conditions is now becoming more and more obvious. It is important to explore this intricate biological coordination and determine the functional mechanisms at metabolic and genetic levels to use the system optimally in stressed habitats. Concerted research toward novel bioformulations for stressed agro-ecosystems, their field screening, improved delivery systems, and higher shelf life, are needed. The use of microbial metabolites and additives such as EPS, biosurfactants, and nutrients need to be explored for bioinoculants developed specifically for saline soils. Engineered nanomaterials are now being considered as next generation carrier materials for the development of tailor made nano-formulations that can work with precision to increase site specific and controlled nutrient availability in stressed soil conditions.

The proper monitoring and mapping of salt stressed habitats, involving as wide as satellite imaging and as narrow as micro-ecosystems in soils (for the level of ions, nutrients, and microbial communities) is also of importance so that these systems can be properly and timely managed. These will provide insights about the existing microbial communities, which are unique in their characters and can be helpful for agriculture in saline areas. Soil engineering through tools like metabolomics and metagenomics can help decipher novel microbial metabolites from the rhizospheric ecobiome. The repeated use of green products will be crucial to increasing soil organic matter, nutrient content, and microbial load in salt stressed soils through the process of rhizoremediation (Figure 1). This Research Topic thus summarizes how the co-evolutionary relationship between rhizobacteria and their host plants can help increase the yield of crops in salt degraded lands and provide new possibilities for exploration and research that can altogether change the future of bioformulations for sustaining the agro-ecosystems in general and for remediation of stressed ecosystems in particular.

**Figure 1:**
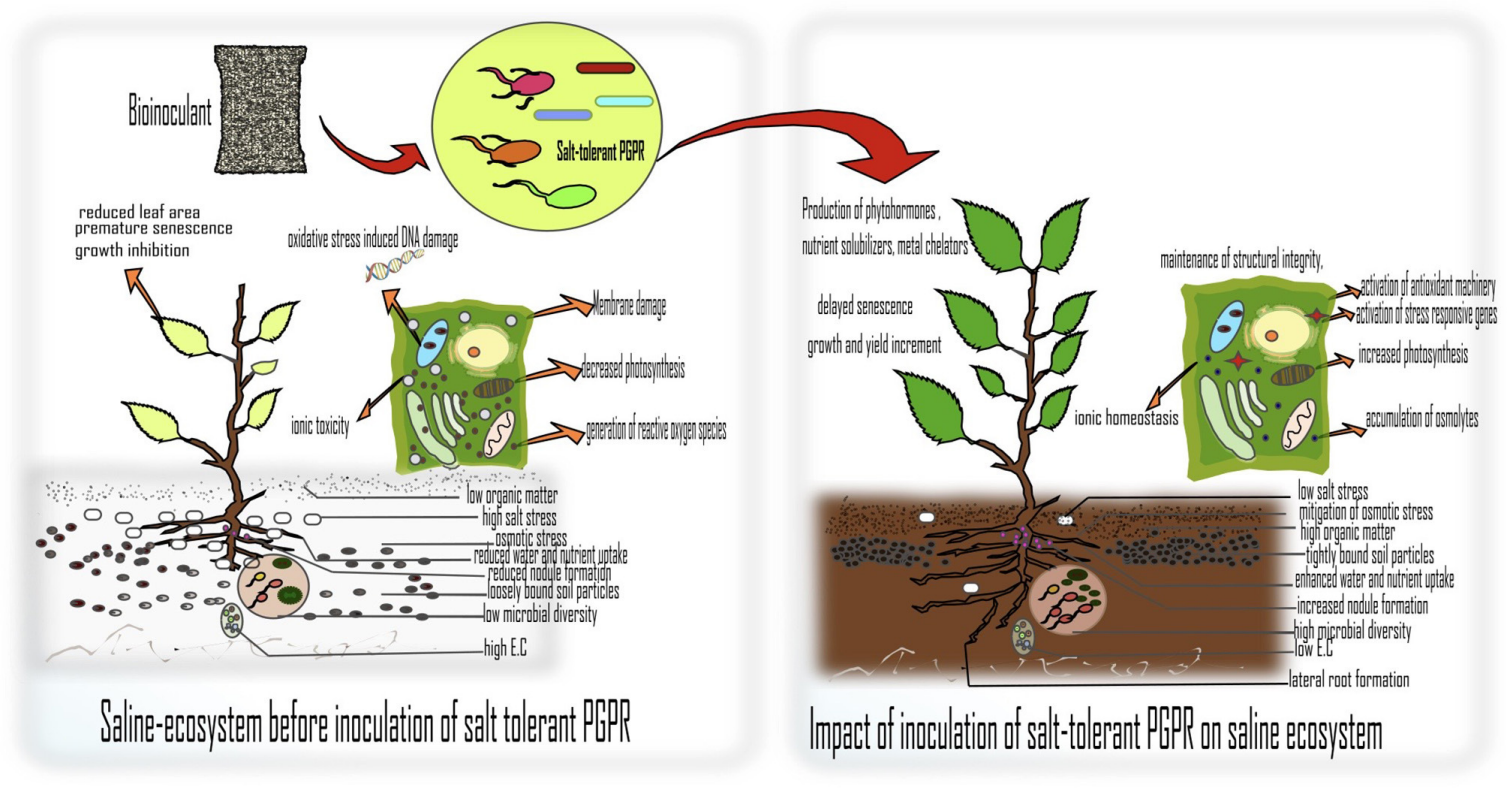
Mechanisms of improving plant health and remediation of saline soils by inoculation of PGPR.

## Author Contributions

NA conceptualized the idea. Figure was drawn by NA and IM. All the authors helped in writing the manuscript.

## Conflict of Interest

The authors declare that the research was conducted in the absence of any commercial or financial relationships that could be construed as a potential conflict of interest.

